# Ruptured left gastric artery aneurysm presenting with life-threatening intraperitoneal hemorrhage: a rare vascular emergency

**DOI:** 10.1590/1677-5449.202502172

**Published:** 2026-09-18

**Authors:** Ayman Hassan Khamis Alserr, Mohammad Salem Amer, Malak Mohsin Almarhoun, Mansoor Cherumkuzhiyil Abdulla

**Affiliations:** 1 Sultan Qaboos Hospital, Salalah, Dhofar, Oman.

**Keywords:** gastric artery aneurysm, intraperitoneal hemorrhage, vascular emergency, aneurisma da artéria gástrica, hemorragia intraperitoneal, emergência vascular

## Abstract

Visceral artery aneurysms are rare, with left gastric artery aneurysms representing a small proportion. Most are asymptomatic and incidentally detected, but rupture can lead to life-threatening intra-abdominal hemorrhage. We report a 50-year-old male presenting with sudden epigastric pain and hypovolemic shock. Imaging revealed a ruptured left gastric artery aneurysm with active bleeding and extensive hemoperitoneum. Emergency laparotomy with aneurysm excision and vessel ligation was performed successfully. The patient’s postoperative course was uneventful, and follow-up imaging showed no residual aneurysm. This case underscores the need for early recognition and prompt surgical intervention to improve outcomes in this rare condition.

## Resumo

Aneurismas de artérias viscerais são raros, sendo os da artéria gástrica esquerda uma pequena parcela desses casos. A maioria é assintomática e detectada incidentalmente, mas a ruptura pode levar a hemorragia intra-abdominal com risco de vida. Relatamos o caso de um homem de 50 anos que apresentou dor epigástrica súbita e choque hipovolêmico. Os exames de imagem revelaram ruptura de aneurisma da artéria gástrica esquerda com sangramento ativo e hemoperitônio extenso. Foi realizada laparotomia de emergência com ressecção do aneurisma e ligadura do vaso com sucesso. O pós-operatório do paciente transcorreu sem intercorrências e os exames de imagem de acompanhamento não demonstraram aneurisma residual. Este caso ressalta a necessidade de reconhecimento precoce e intervenção cirúrgica imediata para melhorar os desfechos nessa condição rara.

## INTRODUCTION

Visceral artery aneurysms are rare vascular anomalies, with an estimated incidence of less than 2% in the general population.^1^ Among these, left gastric artery aneurysms are exceptionally uncommon, accounting for approximately 4% of all visceral artery aneurysms.^2^ Most cases remain clinically silent and are discovered incidentally during imaging studies performed for unrelated indications.^3^ However, rupture of such aneurysms—though rare—carries a high risk of mortality and requires prompt recognition and urgent intervention.^2^ The optimal timing of intervention in visceral artery aneurysms remains a subject of ongoing debate, particularly in asymptomatic cases.^3^ While symptomatic aneurysms and pseudoaneurysms are widely accepted as requiring urgent treatment, management of incidentally detected true aneurysms depends on size, growth, location, and patient-specific factors, as outlined in current clinical practice guidelines.^4^ A further challenge lies in their often nonspecific presentation, with many cases remaining asymptomatic until rupture, which may manifest as acute abdominal pain, gastrointestinal bleeding, or hypovolemic shock.^5^ Although rupture of a left gastric artery aneurysm is rare, it may be considered among the differential diagnoses in patients presenting with unexplained intra-abdominal hemorrhage or sudden hemodynamic instability. In this report, we describe a case of a ruptured left gastric artery aneurysm presenting with hemorrhagic shock, highlighting the diagnostic challenges and management considerations associated with this uncommon but life-threatening condition.

## CASE REPORT

A 50-year-old male with no significant past medical history presented to the emergency department with sudden-onset, severe epigastric and periumbilical pain associated with vomiting. One hour prior to symptom onset, he had been playing with his son, who was jumping on his abdomen. His only surgical history was an open appendectomy performed 20 years earlier. At presentation, he was diaphoretic, hypotensive, and in shock. The patient had no previous history of abdominal trauma, recent surgical interventions, or endoscopic procedures. On arrival, he was in hypovolemic shock, with a blood pressure of 70/40 mmHg and tachycardia. On physical examination, the abdomen was soft but diffusely tender with guarding. Resuscitation was initiated with two liters of intravenous normal saline, resulting in improvement of his blood pressure to 125/75 mmHg. Initial laboratory investigations revealed a hemoglobin level of 10 g/dL, which subsequently dropped to 7 g/dL within one and a half hours. His lactate level was elevated at 4.3 mmol/L, while C-reactive protein, erythrocyte sedimentation rate, and other biochemical parameters remained within normal limits. Contrast-enhanced computed tomography of the abdomen demonstrated an active bleeding point with contrast extravasation into the lesser sac, arising from a branch of the left gastric artery, accompanied by extensive hemoperitoneum. The bleeding focus was contained within a saccular structure of soft tissue density/clotted blood, subsequently confirmed as an aneurysm originating from the left gastric artery (Figure 1). Given these findings and the patient’s hemodynamic instability, an emergency laparotomy was performed. Supraceliac aortic control was achieved, and the lesser sac was opened, revealing a ruptured aneurysmal sac measuring 7 × 5 cm arising from the left gastric artery (Figure 2). The sac was opened, and plication of the aneurysmal ostium was performed, followed by gradual declamping of the aorta. The aneurysm was then excised, and the left gastric artery ligated. Inspection of the stomach and small bowel confirmed viability. A drain was placed in the operative field, and the patient was transferred to the intensive care unit in stable condition. His postoperative course was uneventful. He tolerated oral intake, regained bowel function, and was able to mobilize without difficulty. HIV, venereal disease research laboratory test, hepatitis B, and hepatitis C serology were all negative. Autoantibody profiles, including antinuclear antibody, c-antineutrophil cytoplasmic antibody, and p-antineutrophil cytoplasmic antibody were all negative.

**Figure 1:**
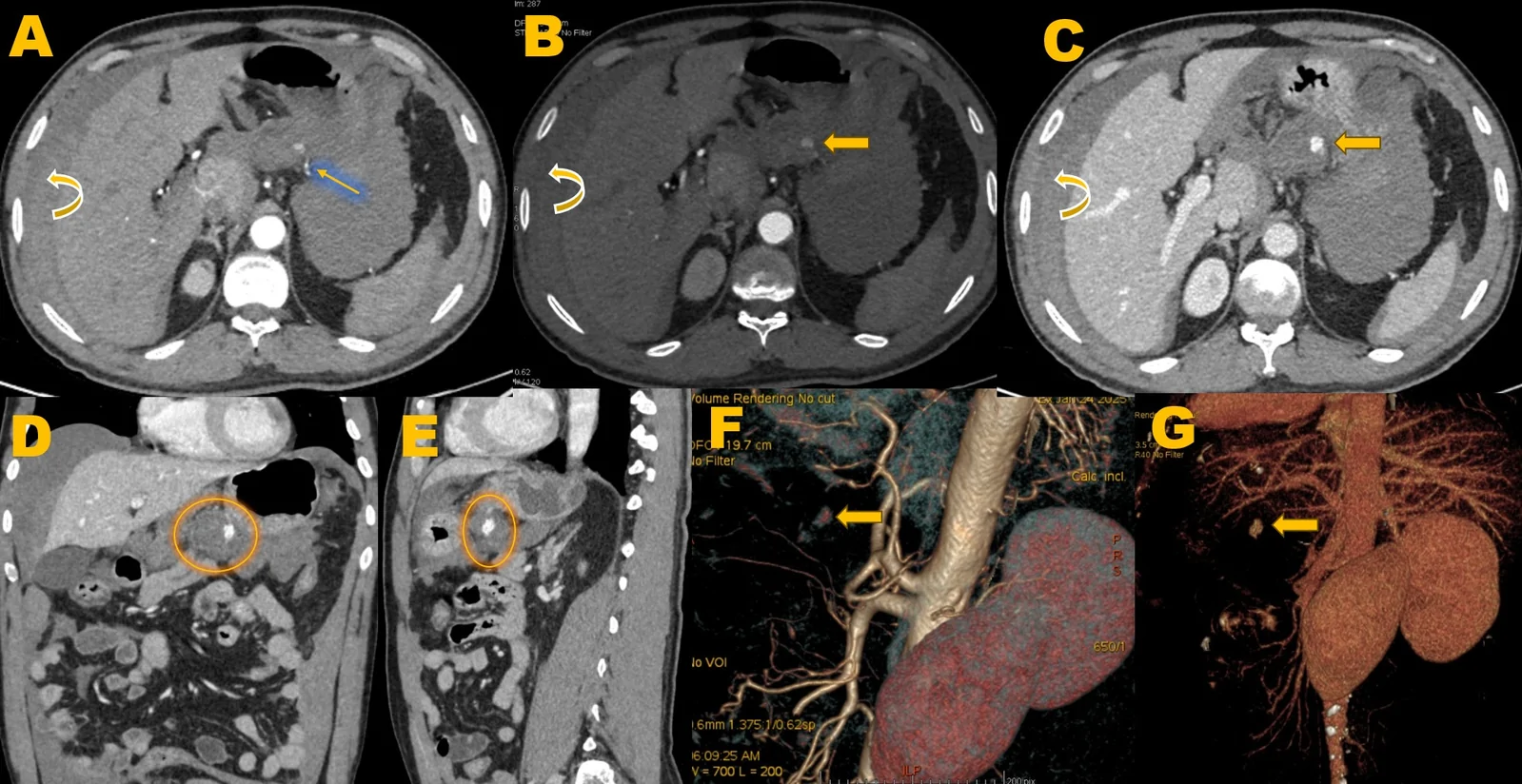
Preoperative computed tomography angiography (CTA) of the abdomen, **(A-C)** axial, **(D)** coronal, and **(E)** sagittal views, along with **(F)** and **(G)** three-dimensional reconstructed images. The images demonstrate an active bleeding point (thick arrow) with contrast extravasation into the lesser sac, arising from a branch of the left gastric artery (thin arrow), accompanied by extensive hemoperitoneum (curved arrow). The bleeding focus is contained within a saccular structure (circle) of soft tissue density/clotted blood, subsequently confirmed as an aneurysm originating from the left gastric artery.

**Figure 2:**
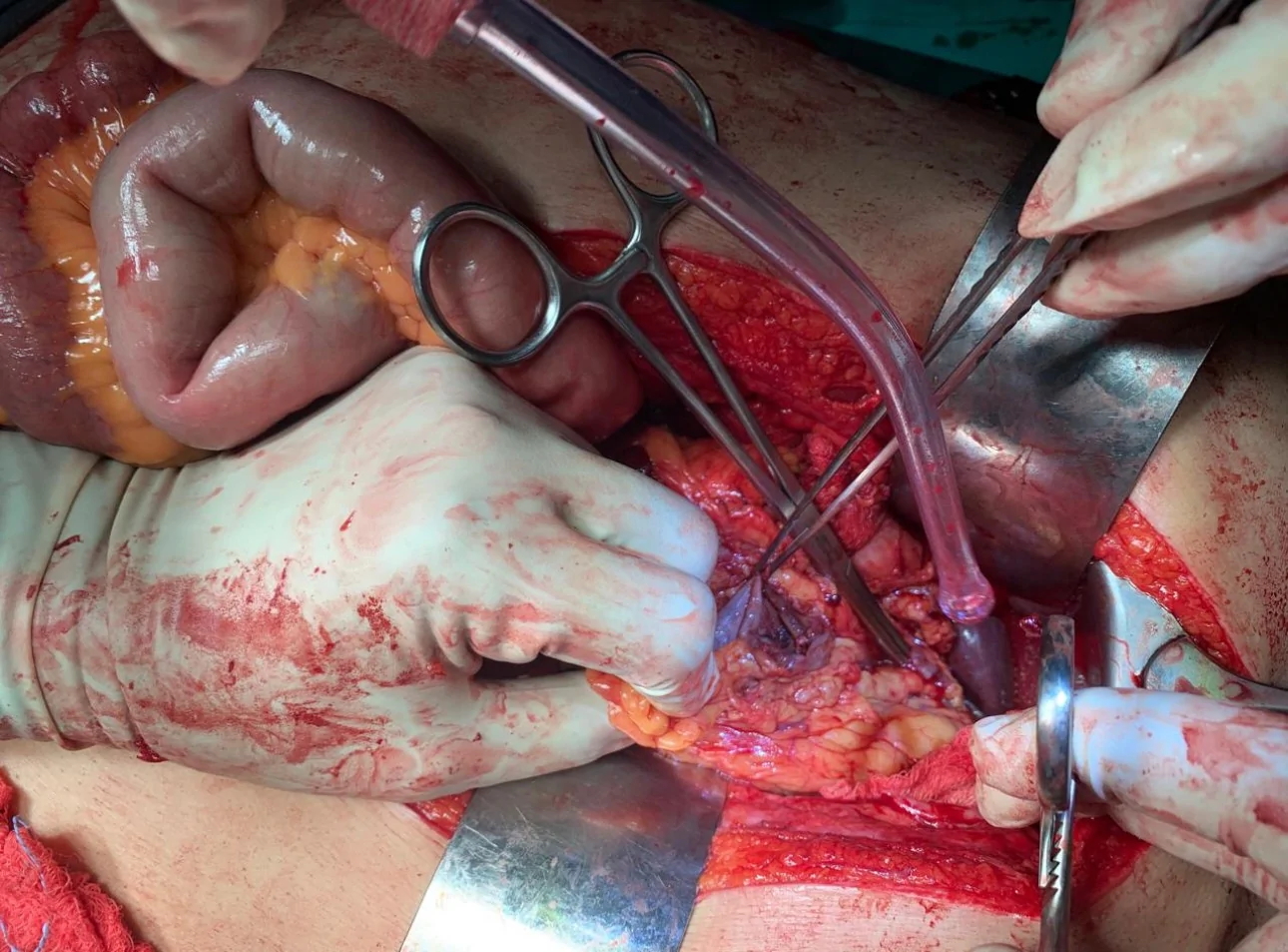
Intraoperative view showing a ruptured aneurysmal sac measuring 7 × 5 cm arising from the left gastric artery.

A follow-up CT scan on postoperative day 1 revealed an organized hematoma at the operative site and another along the greater curvature (Figure 3A). His hemoglobin remained stable at 9 g/dL. A subsequent ultrasound on day 6 demonstrated no significant intraperitoneal free fluid. The abdominal drain was removed, and the patient was discharged in good condition on postoperative day 9.

**Figure 3:**
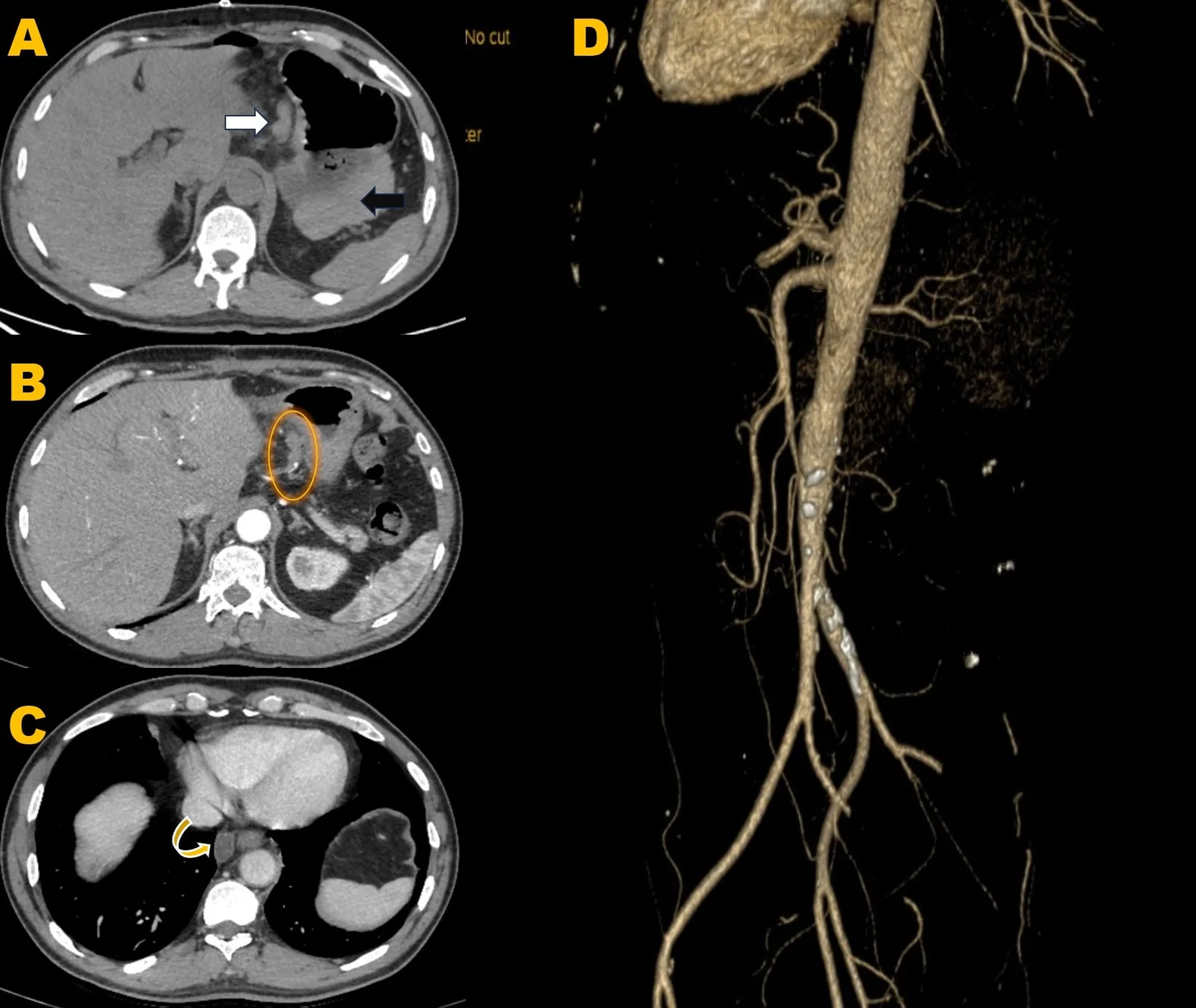
Post-operative abdominal computed tomography (CT). **(A)** Axial cut on the first post-operative day shows an organized hematoma at the operative site (white arrow) and another along the greater curvature (black arrow). **(B)** and **(C)** Axial cuts of a follow-up CTA performed one month later with **(D)** three-dimensional reconstruction reveals a collapsed nodular structure (circle) at the operative site without evidence of re-bleeding, and a small localized collection in the posterior mediastinum adjacent to the lower esophagus, consistent with an aging hematoma.

At follow-up in the vascular clinic after 3 months, a computed tomography angiography (CTA) demonstrated a collapsed nodular structure at the operative site, with no evidence of residual aneurysm or active bleeding. A small loculated collection measuring 2 × 1.3 cm was noted in the posterior mediastinal region adjacent to the lower esophagus, consistent with an aging hematoma (Figures 3B, C, and D). The patient remained asymptomatic and was advised to undergo regular surveillance.

## DISCUSSION

Most cases involving left gastric artery aneurysms present initially with rupture, manifesting as acute abdominal symptoms and hypovolemic shock, as seen in our patient.^6^ In rare instances, patients may report epigastric discomfort, chest pain, or intrathoracic bleeding before rupture.^7^ There is a general consensus that symptomatic VAAs require prompt intervention, as rupture is associated with high mortality rates.^5,8^ Computed tomography angiography (CTA) plays a pivotal role in diagnosis, allowing accurate localization of the aneurysm, detailed visualization of the celiac axis, and differentiation from other vascular or neoplastic lesions.^9^

The etiology of gastric artery aneurysms includes atherosclerosis, inflammatory conditions (e.g., pancreatitis, peptic ulcer disease), vasculitides, trauma, and iatrogenic injury, with pseudoaneurysms more likely in the latter scenarios.^6,7^ Notably, our patient had no identifiable risk factors, making this presentation remarkably unusual. In cases of acute rupture with hemodynamic instability, open surgical management remains the preferred approach.^8^ For non-ruptured visceral artery aneurysms, both open and endovascular approaches are effective treatment options, with endovascular repair offering a less invasive alternative in selected patients. According to the Society for Vascular Surgery (SVS) guidelines, all gastric and gastroepiploic artery aneurysms should be treated irrespective of size, given their relatively high risk of rupture, which does not reliably correlate with aneurysm diameter.^10^ Earlier European Society for Vascular Surgery (ESVS) guidelines applied general size-based thresholds (typically >20–25 mm) for intervention in less common visceral aneurysms.^11^ However, more recent updates have shifted toward site-specific recommendations, aligning more closely with SVS guidance by advocating earlier treatment for high-risk locations such as gastric artery aneurysms.^8^ Overall, current evidence supports prompt treatment of all pseudoaneurysms and symptomatic true aneurysms, while intervention should be considered for asymptomatic aneurysms larger than 2 cm or those demonstrating rapid growth. In rupture scenarios, rapid hemorrhage control is paramount; current guidelines favor endovascular therapy as the first-line approach in patients who are hemodynamically stable or can be rapidly stabilized, provided the anatomy is suitable, due to its lower morbidity and mortality compared with open surgery.

A multidisciplinary approach is essential in the management of visceral artery aneurysms, particularly in acute or complex cases. Close collaboration between vascular surgeons, interventional radiologists, and critical care teams facilitates timely decision-making and optimizes patient outcomes. In addition, structured postoperative surveillance with periodic imaging is important to detect recurrence, residual lesions, or the development of new aneurysms.

During follow-up, arterial-phase cross-sectional imaging is recommended, as VAAs can occur concurrently; in our case, no additional aneurysms were detected, and the postoperative course was uneventful.^4^ For patients with asymptomatic visceral artery aneurysms, annual imaging follow-up is recommended to monitor for aneurysm growth or changes in morphology.^8^

Published reports of ruptured left gastric artery aneurysms consistently highlight their abrupt and often catastrophic presentation, most commonly with hemoperitoneum and hemodynamic instability. Notably, a substantial proportion of cases, including ours, lack identifiable predisposing factors, emphasizing the unpredictable and occult nature of this entity. Across the literature, computed tomography angiography has emerged as the cornerstone of diagnosis, enabling rapid localization and characterization of the bleeding source in time-critical settings. Although there is an increasing shift toward endovascular management in anatomically suitable and hemodynamically stabilized patients, open surgical intervention remains indispensable in unstable presentations requiring immediate and definitive hemorrhage control.

## CONCLUSIONS

Ruptured gastric artery aneurysm, although rare, may be considered in the differential diagnosis of patients presenting with unexplained hypotension and acute abdominal pain. Its often nonspecific presentation and diagnostic challenges necessitate a high index of suspicion and appropriate use of imaging for timely identification. Management should be individualized, taking into account anatomical location, aneurysm characteristics, clinical status, and available treatment options.

### Declaration of patient consent

The authors certify that they have obtained all appropriate patient consent forms. On the form, the patient gave his consent for his images and other clinical information to be reported in the journal. The patient understands that neither his name nor initials will be published and due efforts will be made to conceal his identity, but anonymity cannot be guaranteed.

### Research quality and ethics statement

The authors followed applicable EQUATOR Network (http://www.equator-network.org/) guidelines, notably the CARE guideline, during the conduct of this report. We also certify that none of the authors is a member of the Editorial Board .

### Compliance with ethical standards

All procedures performed in studies involving human participants were in accordance with the ethical standards of the institutional and/or national research committee and with the 1964 Helsinki declaration and its later amendments or comparable ethical standards.

## Data Availability

Data sharing does not apply to this article, as no data were generated or analyzed.
